# New York Pathological Society

**Published:** 1859-09

**Authors:** E. Lee Jones


### ART. II.—*New York Pathological Society.*

REGULAR MEETING, March 9, 1859. JOHN C. DALTON, JR., President.

(Reported for the Journal, by E. LEE JONES, M. D., Secretary.)

### STRICTURE OF URETHRA, ETC.

Dr. Robert Ray presented a specimen of stricture of urethra, removed from a patient of the New York Hospital. The patient, aged 65, had been an inmate of the hospital for the last forty years. He was first admitted with gonorrhœa; this was followed by a stricture and a perineal fistula. He was in the habit of passing the bougie through the opening in the perineum.

At the post-mortem examination there was found a stricture at the junction of the membranous and spongy portions; and a quarter of an inch behind that, was the perineal fistula. The fistula was nearly as large as the normal urethra. The urethra itself was not dilated behind, owing, no doubt, to the free passage into the bladder.

Dr. Post said that the stricture, notwithstanding its age, was a linear one, being confined to a very small portion of the urethra. If such a condition of things could have been diagnosticated, the operation for perineal section would have been successful.

### STONE IN BLADDER, ETC.

Dr. Ray next presented the kidneys, ureters, and bladder, of a man who died at the New York Hospital ten days before. He came in suffering from symptoms of stone in the bladder. The history of the case was in brief as follows: About five years ago, for the first time, he was seized with a severe pain in the lumbar region, which pain only lasted at that time for a few days. Very soon after that, began symptoms of stone in the bladder, stoppage of water, bloody urine, &c. This state of things lasted five years, at the end of which time he entered the hospital. At that time he was suffering a good deal from fever, and he had a good deal of constitutional disturbance. Soon irregular chills came on, and he sank and died ten days after his admission.

On *post-mortem examination* these specimens were found. The left kidney was enlarged, and external to it there was an abscess of very considerable size. The ureters were very much dilated; in some points they equaled in calibre the small intestines. The coats of the bladder were much thickened. There was a small abscess in the perineum, containing about an ounce of pus, which opened into the urethra. There was no stricture of the urethra. In the bladder was found a beautiful mulberry calculus, about the size of a hen's egg.

FATTY TUMORS.

Dr. A Post presented a specimen of a *fatty tumor* weighing over eight pounds, removed from a female fifty-two years of age. It was situated on the upper extremity, and its attachment extended about one-third the distance from the shoulder to the elbow. The tumor was composed of fatty tissue, imbedded in which was a number of calcareous concretions. She had carried the tumor for twenty-six years. The patient recovered from the operation, the wound healing by first intention.

SYPHILITIC SORE THROAT. ŒDEMA GLOTTIDIS.

Dr. Jas. R. Wood presented a specimen of the *larnyx and trachea* of a man sixty years of age, with the following history: About five years ago the patient contracted chancre, which in the course of time was followed by secondary disease of the throat. A few days ago he entered Bellevue Hospital, with stridulous breathing, and cough, and all the physical signs of ulceration about the glottis. On Saturday, at my clinic, the house-surgeon called my attention to him. I made an examination, and told my class that the patient was suffering from syphilitic disease of the throat, with more or less œdema about the glottis. Upon examining the chest, I discovered the physical signs of tubercle. I recommended that he should be placed under treatment, with fumigations of cinnabar, also giving directions that he should not be left alone, as he was liable to die at any moment. The prescription was given repeatedly; but the precaution of close watching was not followed out, for on Sunday morning he took himself to the water-closet, suddenly became faint, was carried to the bed, and found to be dead. He died without any warning.

On making the *post-mortem examination*, there was found to be a good deal of serous infiltration of the sub-mucous cellular tissue in the neighborhood of the glottis, in the cellular tissue, above the epiglottis, and around it; and also a considerable deposit of lymph. The arytenoid cartilages and mucous membrane in the vicinity were ulcerated. This point was œdematous, hung over the glottis, and closed it up.

This case, Mr. President, would have been relieved, and probably cured, if the operation of tracheotomy had been performed. I have cut into eleven tracheæ for syphilitic disease, and only two died; the others all recovered. During my first service at Bellevue, the syphilitic patients were under our care; at that time tracheotomy was looked upon as an almost uniformly successful operation. I recollect two instances where tracheotomy was performed without there being sufficient trouble at the time to warrant it,—with the view, in the first place, of preventing sudden death, and in the second place, of allowing the organs to become passive, that the treatment might be better carried on. I believe it is good practice, and that this man might have been saved had the operation been performed. The sudden death seemed to have been caused by the exertions in the water-closet.

Dr. Sayre stated that the operation of tracheotomy was by no means a serious one, and was of the utmost importance in such cases. In cases of long-standing ulceration of larynx, an operation is followed very often by a rapid recovery. This fact he attributed to rest more than anything else.

### MALIGNANT DISEASE OF LARYNX.

Dr. Wood presented a specimen of *malignant disease* of the *larynx*, from a patient who died in the hospital that day. The patient entered the hospital on the 5th of the month, in an anæmic condition, with some difficulty of breathing, and a slight cough. He was examined by the house-physician, Dr. Jones, who found very slight disease of the lungs. He was placed upon a tonic course of treatment; he seemed to be doing very well until to-day, when the orderly went to his bed and found him dead.

*Post-Mortem Examination.*—Upon opening the thorax, nothing of interest was found; everything seemed to be in a healthy condition; but upon removing the trachea and pharynx, a tumor was discovered about midway between the cricoid cartilage and the first division of the bronchia. On dissecting the parts I discovered a tumor on the right of the trachea, and engrafted so firmly upon it as to become a portion of it; and as a consequence, there is almost complete occlusion of the respiratory tube at that point. The tumor, upon examination, was found to be malignant in its character. The previous history of the case is not known.

Dr. Post asked what was the original seat of the disease.

Dr. Wood presumed that it commenced in the coverings of the cartilaginous rings; there were no lymphatic glands invaded in the neighborhood.

Dr. Wood presented a third specimen of sero-cyst of the supra-renal capsule. It appeared to be connected with the coverings of the capsule, and was about the size of a hen's egg. There was no disease of the kidney whatever.

### DISSECTING ANEURISM OPENING INTO PERICARDIUM. AUTOPSY.

Dr. Clark next presented a specimen of dissecting aneurism. He stated that he had the opportunity of presenting rather an interesting specimen of dissecting aneurism at a late meeting, the interest of the case, perhaps, depending more upon the fact that the heart at one time would cease to beat for a period of fifteen seconds, than on the aneurism itself. He stated that he had an opportunity of presenting a specimen of dissecting aneurism, occurring in the practice of the same gentleman from whose patient the last specimen was taken. Dr. Van Kleek has had two dissecting aneurisms in his practice within the period of six weeks. The history he could not furnish at that time, but he hoped to be able to do so at some future meeting. He had but one single fact to narrate, not having seen the case during life, viz., the mode of death. He did not know the duration of the symptoms of aneurism, but had a vague idea that after the aneurism had formed, the patient lived for twelve or sixteen hours.

During the continuance of these symptoms, said he, there was no pulsation in the left arm. The specimen will show the reason of that, when examined. I will first remark, in regard to it, that the heart is considerably enlarged. The pericardium has no attachment to the organ. The aneurism opened into the upper portion of the pericardium, as in the other case; and about the same quantity of blood escaped into the sac, twenty ounces. The precise point where the opening occurred, I am not able to point out; but it is upon the aorta, at a little distance from the heart, evidently in the pericardial sac, from the amount of ecchymosis that is in the neighborhood.

The point that I wish to call attention to, particularly, was this—that the dissection, by its beginning in the aorta, extended into the large vessels given off by the aorta, and that the cause of the fact that is referred to, the cessation of pulsation in the left arm throughout, was a complete closure of the artery by a clot that had formed in the space made by the dissection. In other words, passing an instrument like this long forceps into the artery, into the aorta, and thus into the large vessels, the innominata and left subclavian particularly, it follows the course of separation. The dissection runs in such a way as to force the inner half of the walls of artery against the wall of the opposite side, and so shut off the channel completely. In that place was a clot that completely filled the artery.

ABSCESS COMMUNICATING WITH INTESTINES.

Dr. Church next presented a specimen of a portion of the walls of the abdomen and intestines, removed from a gentleman who died about two months ago. Early last fall he was seized with an attack of bilious colic; the urgent symptoms were treated, but a diarrhœa followed, which continued for two or three weeks; then the discharges assumed a muco-purulent character. This condition of things continued for two weeks longer, and after that he seemed well, with the exception of pain in the left iliac region whenever he walked fast or moved suddenly. He continued in this state until about six weeks before his death, when a fluctuating tumor showed itself in the left iliac region. It was determined to open the abscess, which was accordingly done, and pus of such an offensive character escaped that it was impossible to remain in the room. It was concluded, from that circumstance, that the abscess communicated with the intestine. The man rallied after the operation ; but soon a cough developed itself, and he sank and died ten days after.

*Autopsy.*—On post-mortem examination, the opening of the walls of the abdomen was found to have a communication with a portion of the small intestine, which was firmly adherent at that point. There were two openings of the abscess into the intestine. The right pleura was filled with about twenty ounces of sero-plastic fluid.

Dr. Wood stated that it was a remarkable fact in this case, that ulceration of the small intestines should have taken place at that point.

Dr. Sayre stated that he had under treatment a patient who was operated upon by Dr. Van Kleek, five years ago, for this difficulty ; and there still remained, in the fold of the groin, a small opening which communicated with the intestine. Through this opening gas frequently escaped.

### CRANIO-TABES.

Dr. Krakowitzer presented a very interesting specimen of cranio-tabes, with the following history—

The child from which this specimen was taken was born on the 19th of last September, and I saw it about two days after, when I called on the mother for trouble of the breast. The father is a healthy man ; the mother, however, is a little delicate, and very hysterical, otherwise she is quite healthy. The child was never a strong one, always pale, never with a very good digestion, and was brought up by Borden's condensed milk. I saw the child, off and on, quite frequently ; it was generally brought to my office. The main complaint was that the child was very costive, frequently going for three or four days without a passage ; as a consequence of this, it would become very restless. I administered simple injections, and occasionally gave rhubarb and magnesia ; a passage from the bowels would be the result, and the child would become more quiet again. It adapted itself pretty quickly to the artificial nourishment, and grew pretty strong, but never showed that exuberance of animal spirit that is common with children. I saw the child on the 19th of January, when I vaccinated it ; the vaccine took its regular course. About three weeks afterwards I was called to attend the child, and found her suffering from a moderate amount of catarrhal inflammation, which subsided, under treatment, in a couple of days. At this time I noticed a peculiar conformation of the child's head. There were four well-marked protuberances occupying the anterior third of the parietal and the posterior half of the frontal bones, divided by a shallow, crucial furrow, in the situation of the anterior fontanelle, and taking the direction of the sutures. At the same time there was a marked obliquity of the skull. The posterior aspect of the right side of the head was considerably flattened, as was also the anterior aspect of the opposite side, making one oblique diameter smaller than the other; that is to say, taking the measurement from the ear on the right side obliquely across to the left frontal bos, the distance was much shorter than on the opposite side. The whole head seemed as if it were twisted, the normal diameters being changed in their relations to each other, without any diminution of the size of the head as a whole; for, while one part, say the right posterior aspect, was flattened, there was a compensating bulging on that same side anteriorly, and on the opposite side *vice versa.*

The existence of this singular conformation of the head led me to make an examination, suspecting it to be a case of *cranio-tabes.* I then found an unnatural yielding of that portion of the skull, on the right side posteriorly, immediately in front of the lambdoidal suture. The area of this softening was at that time about an inch in extent. By pressing upon this point, no reflex action was induced, nor did the child seem to suffer much pain. By making a pretty firm and steady pressure on either of the protuberances just described, a mark was produced, which it took some time to obliterate. This pressure obviously caused more pain than that on the thin part of the skull.

On no other part of the skeleton could any abnormal softening or deviation from the natural shape be detected. The chest was finely formed, and by auscultation and percussion nothing abnormal could be elicited. The child could lie on either side, and seemed to be well nourished, only the skin had a paleness like marble. There was, besides this, a slight puffiness of the feet and hands.

The diagnosis of cranio-tabes of the posterior part of the skull, with the usual eccentric hyperostitis of the anterior part, being very clear, a tonic treatment was commenced. The patient was given the compound syrup of superphosphates ʒ ss, twice a day, and for nourishment half a pint of Borden's condensed milk, and beef-tea as much as one pound of the best beef would give.

The child took, up to three days before its death, the nourishment very readily, always the whole allowance of milk, and generally the beef-tea. Some days, however, the appetite would vary. The sleep was fair, though the child would wake up four or five times of a night; still it would make up for this want of rest by sleeping a considerable part of the day. There was generally only one normal passage of the bowels in twenty-four hours, and it was of a light color.

The urine was examined about the middle of March; only an ounce could be collected in three days, though the normal quantity seemed to be passed. The urine was found to be turbid, and of neutral reaction. On the application of heat it remained turbid, but cleared up on the addition of nit. acid.

The percentage of inorganic salts was only one quarter of the normal quantity. The symptoms gradually progressed, the softening of the bone alluded to becoming increased in extent. At one or two points on this portion there could be recognized defects in the bony structure by the finger-nail; abrupt margins could be detected that seemed as if a small hole existed in the bone.

On the 23d of February Dr. Jacobi saw the case with me, and corroborated all the facts that I had made out. At that time there was no deficiency of the bony parts of the skull noticeable except the portion referred to in front of the lambdoidal suture.

Dr. Gouley saw the case with me on the 3d of March. On the 9th of March, for the first time, I noticed that there was a small spot on the left side of the occiput that did not resist pressure; in other words, the bone was softened at that point. As the case progressed, I could, by pressure with the finger-nail, appreciate the entire absence of the bony structure at one or two points. By observing this dried specimen, the bone at such points is found to be entirely absorbed, the openings being closed over by the pericranium and dura mater, which I have left attached.

On the 19th of March I noticed for the first time a petecchial eruption over the right arm.

As the case progressed I noticed that the liver began to enlarge sensibly, and continued to do so until the time of death. This fact is, perhaps, of some importance, when we take into account the emaciation notwithstanding she seemed to take nourishment very well. The skin was pleasant during all this time, and had not the dry, harsh feel that we notice so frequently in marasmus. From time to time I would notice a puffiness or œdematous swelling of the hands and feet; the petecchial eruption would go and come, at longer or shorter intervals.

Dr. Gouley, at the time he saw the case, coincided with me as regarded the plan of treatment. At the same time, he suggested the propriety of administering the syr. iod. ferri in addition to the other remedies. I accordingly gave this along with the syr. of the super-phosphates.

On the 1st of April strong febrile action set in; the stools, changing their character, became frequent, and consisted merely of undigested milk. The syrups were then discontinued, and alteratives were given with a view to stimulate the action of the liver, but to no purpose. She died from exhaustion, without convulsions, April 4th, at 7 P. M.

The post-mortem examination was made sixteen hours after death. The connecting tissue of the scalp was very œdematous. The brain was very pale throughout, and also very œdematous. There was no flattening of the convolutions. The lateral ventricles were not distended. The lining membrane of the ventricles was not softened. The pia mater and the choroid plexus were very pale. On examining the skull we found it to be of this oblique shape, as I have already described. The bones, except at those points where absorption had taken place, were unusually thickened, but very succulent. The thinning of the skull extends on the right side from the juncture of the anterior and middle third of the parietal bone backward, a little beyond the lambdoidal suture. On the right side it is confined exclusively to the occipital bone, in the neighborhood also of the lambdoidal suture. The vitreous table is normal in appearance, as we find it in children of this age, except at those points where the thinning of bony structure is apparent: there we observe the *impressiones digitatæ*, as in the adult subject. This shows that the convolutions of the brain, pressing against this internal table, has caused absorption of the softened bone, amounting in some spots to a complete deficiency of the bony structure. The only covering for the brain at these parts is the pericranium and dura mater. The pericranium covering the described protuberances was exceedingly vascular, and quite blue in the fresh specimen. This vascularity at the margins of these protuberances, is abruptly arrested, and we see healthy bone. On cutting into one of these protuberances, the knife readily entered into the subjacent tissue; and on dissecting up carefully the flap from the external table, we found that a bony deposit had taken place between the periosteum and the outer table. From the place where the flap was dissected up, there was noticed a peculiar porous condition of the surface of the bone. This is the commencement of a transformation of the external table from the compact to spongy structure; and this, becoming incorporated with the new deposit, and also with the diploë, would leave the bone without a compact layer, if that part of the new formation nearest to the periosteum was not organized into a dense tissue. If life had been prolonged, the diploë would have been very much increased in diameter; this is a fact that has been noticed in similar cases that have been reported.

No other parts of the body were allowed to be examined.

The dimensions of the anterior fontanelle were as follows:—Longitudinal diameter was one and seven-eighths, while the transverse was one and a half inches. The posterior fontanelle was almost closed.

Dr. Krakowitzer, in answer to a question from Dr. Clark, stated that he thought it was a disease of the general system, from the fact that children laboring under this difficulty are never healthy. He did not think it should be compared with rachitic disease; because it occurred mainly in the head, and there was a corresponding increase of osseous matter that was thrown out in the vicinity. In rachitis, bony matter was wanting. There is not a want of the earthy bases of the bone. It has been found, that the disease occurs in a part of Germany where the drinking-water contains a good deal less of lime than it generally does; and that right near, where the chemical constitution of the water is different, the disease does not occur. I am inclined to the opinion that it is a local disease, a disease of the capillary vessels of the skull; that the bone, by some process, gets softer, and that the brain pressing upon the bone, causes it to be absorbed, while new bony matter is thrown out in the vicinity. The new bony matter thrown out contains about thirty-two per cent. of the phosphates; but the bones of the skeleton generally contain the same amount of earthy substance. The convolutions of the brain were not flattened in this case.

Dr. Clark, in this connection, stated that he succeeded very well in collecting the urine in children, by squeezing the diapers. It answered every purpose, for both chemical and microscopical examination.

### PHOSPHORIC DISEASE OF LOWER JAW.

Dr. Markoe presented a specimen of phosphoric disease of the lower jaw, for Dr. Parker, with the following history:

The patient, a German, 22 years of age, had been working in a match factory for eight years, during which time he enjoyed perfect health, until four months previous to admission. At that time, he complained of a vague pain about his teeth, and had two or three extracted. The alveolar processes did not heal; and the bony surfaces remaining bare, seemed to increase in extent. A short time after he noticed this, his face began to swell, and it was soon followed by the formation of an abscess. At the end of two weeks after, the abscess opened itself behind the ramus of the jaw. A second abscess soon after formed, opening underneath the jaw; at the same time, a fœtid discharge began to make its appearance from the inside of the mouth. The case, from that time, progressed slowly and steadily, until he came into the hospital. He then presented an unusually distorted countenance. The external surface of the tumor felt exceedingly hard, so much so that it was thought there must be an involucrum present. Behind the ramus of the jaw, there were the two openings referred to. The operation for the removal of the diseased mass was performed by Dr. Parker. There was nothing peculiar about the operation; the incision was made in the usual place, and the soft parts were raised from the bone, the condyle disarticulated, and other sections at the symphysis menti made by the chain saw. The diseased mass removed presented a beautiful specimen of what Stanley terms pumice-stone excrescence. Nearly the whole surface was covered with this material; the deposit was more abundant, however, at the angle of the bone. The patient did very well after the operation; and after he had remained in the house about a month, new bone had commenced to be deposited in place of that portion which had been removed.

Dr. M. stated, in conclusion, that Van Vebre and Geist, who describe the affection more perfectly, state that the pumice-stone formation only occurs in the lower jaw.

Dr. Van Buren stated that he had seen this same material on other bones.

Dr. Wood thought that it was the result of an aborted growth of bone, that nature throws out in the reparative process, and was peculiar to phosphoric disease.

Dr. Van Buren did not think that the disease was essentially periostitis. It seemed to him that, in this disease, the bone was killed almost immediately. He did not believe that we understood the initial lesion.

Dr. Wood stated that the symptoms were all those which characterized an inflammation of the part passing on to a chronic form; and, unless the surgeon interfered, the patient would die of suppurative inflammation.

Dr. Post was inclined to the belief that the disease was osteitis rather than periostitis.

### FIBROUS TUMORS OF UTERUS.

Dr. Van Buren next presented a specimen of fibrous tumors of the uterus, for a candidate.

Frances H.,—mulatto, aged 50 years, native of Virginia. Phthisis in the family. Was vigorous in childhood. Menstruation before marriage was scanty, painful; had hysteria. About her twentieth year, had hepatic difficulty. Married twice: first time, she was 24 years old; husband died with scarlatina three months afterwards; married again in three years; *no conception at any time.* She noticed the uterine growth commencing in the right side, three years after the last marriage, then 30 years old. Constipation existed previously; the growth did not materially increase it, defecation was performed without great difficulty or pain. Micturition not unnatural until within the last three years. Had menorrhagia; at the periods had great pain and constitutional disturbance, in the interim not much pain Had rheumatism first fourteen years ago, with cardiac complication. Swelling of the lower extremities noticed four years ago, afterwards in the abdomen, and then she became more or less anasarcous. The 3d of last October, she (without any irregularity or prodromic symptoms) lost motion of the left side, and sight, and articulation; appeared rational; not comatose.

Her treatment was general, according to symptoms; about a week afterwards recovered her sight and articulation. Diuretics being of no avail, orthopnœa being such, on account of the fluid, tapping was performed on the 19th ; 1½ pails of fluid were removed ; no unfavorable symptoms followed. Iodide of potassium and oil of juniper, proved good diuretics, subsequently ; paralysis was considerably relieved, she could raise her hand to her face, and her leg. A month previous to her death, fluid accumulated, but not to justify removing it, and the paralysis became aggravated. Died, April 10th, 9 P. M.; nothing remarkable about her death.

*Post-mortem*, twelve hours after death. Body warm; rigor mortis marked on the right side, none on the left. Was not permitted to examine the brain. Over an inch of adipose tissue over the sternum, and more over the abdomen. Right lung filled its cavity, on account of adhesions; the left encroached on partially by fluid; no tubercular deposit observed. Heart hypertrophied, weight twenty ounces; considerable fluid within the pericardium. Liver and kidneys fatty. Uterus with its appendages weighs 4¼ pounds.

At the request of another physician, I tapped the above patient, and made the post mortem; was not connected otherwise; this will account for the want of detail of the symptoms of the case.

Dr. Finnell next presented several specimens: a mass of hydatids, expelled during the fourth month; an hypertrophied heart, with atheromatous deposits in the large vessels, taken from a negro; a uterus, with ovarian cyst; also a uterus of a girl, nineteen years of age, who was brought to the city prison, vomiting, and in a state of collapse, and died. The uterus showed a clot in the interior, with a corpus luteum developed in one of the ovaries. The mucous membrane of the stomach was peeled off in several places. There was no redness or inflammatory action present in the parts.

### Regular Meeting, April 27th.

Dr. Post presented a specimen of *anchylosis* of the *knee-joint* which had been removed some months before, together with a cast of the knee exhibiting the appearance of the part at the time of operation. The patient was a young man who for many years had bony anchylosis following an injury of the joint, the limb being bent at a somewhat acute angle. The operation was performed according to Dr. Buck's method. Great trouble was experienced after the operation, from the excessive tension of the tendons of the flexor muscles. When the patient recovered from the effects of the ether, there was a disposition to violent muscular action. In consequence of this agitation of the limb a considerable amount of hemorrhage took place, sufficient to cause a good deal of distension of the wound, and necessitate the removal of two or three sutures. This was followed by an undue amount of suppuration. Fourteen days after the operation the patient was attacked with tetanus, and died at the end of fourteen days after. Profuse suppuration continued up to the time of death. He observed that the case was an unusual one in relation to the duration of the tetanus; it was very unusual to see death take place so long after the disease manifested itself. Under such circumstances they were very apt to recover. The ends of the femur, tibia, and fibula were consolidated together, forming a firm mass.

Dr. Bauer asked if the muscles were divided.

Dr. Post said that they were not.

Dr. Sayre thought that the division of the tendons would have prevented this spasmodic action.

Dr. Post, in answer to Dr. Bauer, stated that the twitching of the muscles was most marked in the first two or three days.

Dr. Bauer presented a specimen of disease of the hip-joint, with the following history.

*Hip-disease of the third degree ; almost entire destruction of the ligamentum teres ; perforation of the joint ; consecutive abscess below the fascia iliaca, communicating with the latter ; circumscribed abscess in front of the spine ; tuberculous deposit in both kidneys and oviducts, and gray tuberculous infiltration of the mesenterial glands ; death and autopsy.*

This most interesting and equally instructive case, refers to a girl aged fourteen years, who entered the Long Island College Hospital on the 12th of March, *a. c.* Very little could be ascertained as to the health of her family, but that her father died from the effects of injury, that her mother was subject to rheumatic attacks, and that one of her younger brothers had a sore leg and occasional cough, whereas the rest of her brothers and sisters (seven in number) enjoyed perfect health.

Sarah Barthelmess herself, had been delicate since infancy, and had suffered from various diseases. Thus her sexual development had been rendered imperfect, more especially as to menstruation, which had not as yet appeared, whereas the mammary glands were enlarged and the mons veneris covered with hair. She was of dark complexion, rather yellowish, and almost *bronzed*, and her countenance bore the evidences of much and painful suffering, much aggravated in appearance by the gray shade of her deeply seated eye-balls ; skin dry, and nutrition very low, with decided loss of flesh ; digestion much disturbed, furred tongue, want of appetite, slight tympanitis, and irregular evacuations ; constipation prevailing ; some fever, with heat, thirst, and headache ; excited action of the heart, with the usual sound of chlorosis; blood decidedly impoverished (anæmic); lung resonant and respiratory murmur normal.

Her chief complaint, however, was her right hip-joint, being intensely painful on motion, contre-coup, and touch. Pelvis elevated, femur adducted, everted, and shortened by about two inches. The flexion of the thigh proved, under chloroform anæsthesia, to be voluntary. Abduction could not be executed without moving the whole pelvis on the left hip-joint, the adductor muscles resisting permanently, and feeling abnormally tense.

About the joint there was some puffiness, a suspicious kind of serous infiltration, so frequently indicative of a deep-seated abscess; yet fluctuation could not be discovered, except posteriorly to the joint, arising from the latter itself. Though the spine presented the usual, simple lateral curvature appertaining to the obliquity of the pelvis, yet on percussion no pain was manifested; nor was there any structural change discernible in the pelvic cavity.

As to the commencement of the affection, the patient stated that she had sustained a fall upon her right hip some eight months ago, causing immediate and successive pain, with subsequent deformity and shortening of the extremity, rendering the use of crutches necessary. In the mean time she had been placed under the especial care of a physician at Chicago, and underwent a treatment of three months' duration, yet derived no benefit therefrom. After using domestic remedies for some months, and growing worse all the time, she was advised to proceed east, and thus she came under my charge.

As to specified diagnosis, I could not take the case for anything else but for the third degree of hip-disease, with opening of the joint. But the fact that eversion of the extremity was still maintained, proved to my mind that the aperture of the articular cavity could be but a small one, and that some liquid was retained. The mobility of the joint being thus restricted, it was of course impossible to ascertain the condition of the ligamentum teres. As to the nature of the articular contents, I strongly suspected it to be purulent, although no crepitus could be established, nor was there external fluctuation. Yet her vitiated constitution, the duration of the disease, and the violence of the inflammatory symptoms, admitted no doubt as to the existence of suppuration.

Under these circumstances, the prognosis was utterly hopeless. The disease had long since lost its local and traumatic character; it had become thoroughly constitutional,—a complication with which treatment would grapple in vain.

Ordered generous diet, with ale and milk; and directed iron, quinine, and cod-liver oil to be administered. At night, solutio morphiæ acetatis in extr. belladonnæ, to secure rest.

At my next visit I learned that the patient had passed a restless night, having frequently shrieked so loudly as to disturb her fellow-patients. Fever on the increase, and appetite indifferent. Ascribing in part the constitutional disturbances to the nocturnal pains, I decided upon and executed the division of the retracted adductor muscles, and had two cups applied over the joint, after which the patient was placed in my wire apparatus.

This seemed to give her some rest and comfort. She slept evidently better during the succeeding four nights, her disposition improved, and she evinced some desire for food, yet the pulse remained accelerated, feeble, skin dry, and urine saturated. No importance could be attached to the improvement.

On the 5th day fever increased, preceded by a chill at noon, and accompanied by intense headache; assuming now more distinctly the character of a bilious typhoid, with low delirium, and hypostatic pneumonia. Her sufferings terminated on the eleventh day of treatment.

*Autopsy.*—Lungs hypostatically engorged, more especially their dorsal portions; the right apex with a few scattered yellow tubercles. The corresponding pleural lining thickened, slightly covered with plastic material, with some faint attempts to adhesion with the costal portion. Along the spine the pleura was much intumesced and opaque, their cavities containing a moderate effusion. On removing the thoracic viscera, an abscess was unexpectedly discovered in the posterior mediastinum, right in front of the spine, and between the ligamentum longitudinale anterius and the vertebræ, commencing about at the third and terminating blindly at the ninth thoracic vertebra, all being denuded. The heart was well developed; the walls of left ventricle moderately hypertrophied, yet no material impediment or traces of endocarditis. Right ventricle filled with a post-mortem, fibrinous clot, extending into the auricle and pulmonary artery.

Liver in a state of moderate fatty degeneration, and not enlarged; spleen of ordinary size and appearance; mesenterial glands considerably enlarged, and infiltrated throughout with gray tubercular substance. The uterus comparatively small, and oviducts filled with yellow and round tubercles, so as to give them the appearance of ova. Both kidneys show circumscribed tubercular deposits, with surrounding nephritis; *supra-renal glands eminently developed and indurated.*

On examining the right hip-joint, I found the cartilages and synovial membrane pretty well preserved, though changed in appearance, being more white and opaque. The ligamentum teres, with the exception of a few fibres, entirely destroyed; and, around its insertion at the caput femoris, the cartilage just about to detach itself from the subjacent bone; the cavity filled with inspissated pus, and the furca acetabuli, with a gelatinous substance. No part of acetabulum or caput femoris denuded so as to cause a crepitant sound. Superiorly of the acetabular insertion of ligamentum teres, there is a small fissure that passes under the labrum cartilagineum upwards, and connects with a flat sub-iliacal abscess in the fossa iliaca. The contents of this abscess, measuring about ℥ iij, are very thick and yellow, and seem to have lost a good deal of its fluid portion by absorption. The microscopical examination confirmed the renal and mesenterial deposits as tubercular. Neither the pus in the thoracic, nor that of the iliacal abscess, revealed the so-called tubercular cells; whereas the pus corpuscles, which had in part undergone fatty degeneration, were roughened, wrinkled, and evidently in process of regress.

The cartilaginous covering of the affected joint exhibited, under the microscope, the peculiar changes characteristic of incipient inflammation of that structure. Some objects presented the cartilaginous cells highly granulated and enlarged toward the articular surface; in others, their transformation into fibro-plastic cells and direct fibres were noticed, but no pus. If not deceived by the microscope, I have also observed elongated cellular bodies in the greatly thickened synovial membrane. In fine, I subjected the head and neck of the femur to a careful examination. Neither its consistency, color, nor structure indicated the presence of tubercular elements; the microscope failed also to reveal them.

In connection with the present case, the question offering itself for discussion is:—In what structure did the disease of the joint originate?

It was stated in the ætiology, that the joint had been attacked by pain immediately after a fall some eight months ago; and it must therefore be inferred that it was previously intact, and that the fall was the remote cause of the malady. Another fact is, that the structure pre-eminently affected was the ligamentum teres. It strikes me that this ligament is most exposed to be contused, in falls upon the large trochanter; but it is also by virtue of its vascular endowments more susceptible to the inflammatory process, than, comparatively speaking, any other structure of the hip-joint. It is another well-ascertained fact, that in almost all somewhat progressed cases of hip-joint disease, the ligamentum teres is invariably destroyed. I have at least found it so; and Gurth, in his admirable work on the pathological anatomy of articular diseases, has enunciated the same from his observation and by numerous quotations. There can be no doubt that not every case of hip-joint disease originates in the same way. It may arise from periostitis, from diastasis, from tubercular deposits in the cancellated tissue of the caput femoris, and so forth. Yet in the present instance, the ligamentum teres seems to have been the primitive seat of the disease.

There is another interesting point at issue, namely, that hip-disease may exist in a decidedly tubercular constitution, and be remotely influenced thereby, without being directly complicated with tubercular deposits. To be sure, the matter of the joint and iliacal abscess contained an abundance of small granules, but no entire tubercular cells; and the former may therefore have originated in the blastema itself, or be the effect of broken up pus-cells. At any rate, I found no conclusive evidence of tubercular elements in said pus.

The same must be said of the pus contained in the thoracic abscess, which had no direct connection with the malady of the hip-joint, neither anatomical nor causal. That the diagnosis remained imperfect as to this complication cannot be surprising, as there was no symptom whatsoever indicating it. The superficial disintegration of the interested vertebræ renders it highly probable that the abscess was the result of periostitis.

Another singularity is the preference of the tubercular deposits for the kidneys, Fallopian tubes, and mesenterial glands—to the lungs, which, according to statistics, are the chief seat of attraction for tubercular infiltration.

In fine, I should take no exception to the opinion of pyæmic poisoning in Sarah Barthelmess' case, in place of bilious typhoid, since both have great similarity.

Whether the bronze color of the patient had any causal connection with the evident changes of the supra-renal capsules, I am not prepared to say, since this color has proved to be no pathognomonic symptom of that disease; it being sometimes observed in other diseases, and as often absent in affection of that organ.

Dr. Sayre was hardly inclined to look upon the specimen as one of tuberculous or strumous disease of the hip-joint. On examining the joint carefully he noticed, that there was a small portion of the surface of the acetabulum fractured off. He thought that the fracture was the cause of the abscess of the hip,—that it was not a genuine case of morbus coxarius.

Dr. Bauer stated that even if it was a fracture at that point, it would not interfere with the diagnosis in this disease. He did not deny the possibility that diastasis, or fracture through the joint, was the cause of hip-disease; but he did not think that a fracture had taken place at that point.

EXTENSIVE INTERNAL CANCER.

Dr. Bauer presented *specimens* of *cancer of the internal organs*, &c.—with the following history:

The patient referred to was born of healthy parents in Canada, and he was in the enjoyment of perfect health up to within four months of his death. He was of strong constitution, of very active habits, above medium size, weighed 155 lbs., father of three healthy children, and thirty-five years of age, at his death. About ten years ago, he contracted syphilis, but thought himself well treated and entirely relieved. At any rate he was ever since free from secondary symptoms. Two years ago he contused his left testicle by running with some vehemence against the pole of a carriage; traumatic inflammation ensued, but gradually disappeared through rest and other appropriate remedies.

There remained, however, a moderate induration of the epididymis, which gave no inconvenience. About four months ago, the latter became slightly sensitive, and grew rapidly to the size of a large orange. It was, en weeks ago, removed by a surgeon of New York, when the wound closed kindly. The attendant seemed to have been fully aware of the malignant character of the growth, and the microscope is said to have confirmed his conjecture.

Soon after, the disease appeared in the chord, and ran upwards to the inguinal canal, into the abdominal cavity. In this condition the patient placed himself under my charge, on the 16th of March, *a. c.* Realizing at once the precarious character of the malady, I requested the consultation of my colleagues, Drs. Ayres and John Byrne, who confirmed my supposition *in toto*. The treatment was therefore limited to the administration of morphine and belladonna dissolved in aqua lauracenesi; with which I succeeded in allaying pain, and to render the patient comparatively comfortable, despite the rapid extension of the disease.

At our consultation, the liver though slightly enlarged, seemed not to be affected to any great extent; the tumor in the abdominal cavity had not yet made its appearance. The lungs were also intact, in so far as physical signs could be relied upon. Yet, a fortnight before death, the former were quite distinct, and their nodules could be plainly discovered. A week later, the right lung became impermeable to air, whilst the left lung performed still its functions tolerably well. At last this became involved also, first with ronchus crepitant, and the resonance was soon lost, but never to the extent of the right lung. On account of the right lung and liver, the patient had to lay constantly on his right side; and even in this position his respiration was exceedingly difficult and laborious. The heart seemed to have been the last organ affected; for no abnormal sound could be noticed but three days before death; the latter ensued under the additional symptoms of peritonitis, subsequently evidenced by postmortem appearances.

The present case sets a striking example to the established fact that encephaloid disease of the testicle, when removed, reappears and develops with great rapidity in other organs, causing early death of the patient.

Cancer of the heart is comparatively rare, and is mostly the result of extensive cancerous degeneration. In this case the encephaloid of the right ventricle seemed to have originated upon the endocardium, and is connected with other endocarditic appearances, including the tricuspidal and mitral valves. The liver had formed adhesions with the peritoneum; the destruction of which, on removal of that organ, caused the exposure of its parenchyma on its superior surface. I need hardly state that the cancer cells were very abundant and finely developed.

In answer to a question from Dr. Krakowizer he stated that the tumors of the heart were not examined by the microscope.

Dr. Krakowizer did not think that the tumors upon the smooth surfaces of the endocardium were cancerous; they seemed to be nothing more than fibrous deposits. There seemed, however, to be the commencement of cancerous degeneration upon the free surface of the mitral valves.

### EXTENSIVE AMYLOID DEGENERATION.

Dr. Bauer exhibited a third specimen, viz. Extensive Amyloid Degeneration.

A female patient, refusing to give her name or residence, entered the Long Island College Hospital, on the 6th of March, 1859, at half past 4 o'clock P. M. Without assistance she walked up stairs, and took possession of the bed assigned to her. She stated that she had once miscarried, and that she was again in the seventh month of pregnancy. All she complained of was cutting pain in her abdomen, with vomiturition; for which, the house-surgeon administered an anodyne.

A few minutes afterwards she was taken with a chill; and when the nurse returned with a warm cup of tea, she found the patient dying.

Her reception into the institution and her death, took place within less than an hour.

The commission of a crime being suspected, the coroner directed a postmortem examination; in which I was ably assisted by Dr. Ostrander, and the following notes were taken:

### EXTERNAL INSPECTION OF THE CORPSE.

The body is that of a well-developed and muscular woman, apparently some thirty years of age, measuring five feet three and a half inches. No abrasion about the lips, nor any mark of injury or violence. Mammary glands and nipples considerably developed; the latter discharging a milky fluid.

Abdomen largely distended by a firm, uneven body occupying the front, rising from the pelvic cavity to four and a half inches above the umbilicus, and extending three and a half inches below the xyphoid process; its widest part being nine inches; presumed to be an impregnated uterus.

Sexual organs largely developed; the lower portion, about the posterior fourchette, covered with bloody secretion, partly dried upon the integuments.

The whole surface of the body greatly discolored, more especially the neck, right arm, and side of thorax. The color is red-brown, blue-brown, and about the right shoulder almost black.

The subcutaneous veins everywhere much distended, and still darker, so as to be easily traced. The epidermis peels readily off, more especially at the pudendum. On turning the body, the same state of things was noticed, in addition to the largely developed nates.

*The cavity of the cranium* was then opened.

On removing the scalp, the integuments were found to be much engorged with venous blood, which issued also from the venous emissaries.

The posterior portion of the skull was discolored, from its great vascularity; sinuses not unusually filled; brain but moderately congested, otherwise healthy; at the basis cranii small amount of bloody serum, derived from the spinal canal; external and internal jugular veins distended. In front of the larynx a large-sized goitre. Tongue and oral cavity neither discolored nor corroded. Around the rima glottidis, hæmorrhagic discoloration, extending towards the basis of the tongue, with softening of mucous membrane. Pharynx and œsophagus, down to the stomach, paler; mucous membrane œdematous and thickened. Coats of the stomach evidently thickened, around the cardiac, discolored into a brown-red hue. Differently sized hæmorrhagic spots over the whole internal surface, and below the mucosa, which easily peeled off; contents of a pinkish color, creamy, homogeneous, opaque, measuring little more than an ounce.

Both stomach and its contents reserved for chemical analysis.

Liver of considerable size, of light-brown color, and so soft as to break down by its own weight.

Spleen of usual color, likewise enlarged and softened; the remaining portion of the intestines, had not much changed, exhibiting, however, scattered and small hæmorrhagic extravasations.

Abdomen contained about one pint of bloody serun, but no marked signs of inflammation, and especially no pseudo-membranes nor plastic lymph. All venous vessels highly injected and distended. Kidneys enlarged, greatly engorged, capsule readily peeling off and softened. When split, they exhibited the corticle substance to be dark with interspersed, white granules; medullary substance brown-red. Nothing noticeable in ureters and bladder; latter empty.

After a careful removal of the sexual organs, the vagina was first opened, and found of a gray, dark color, more intensified about and near the os uteri. Neck of uterus exceedingly soft, distended so as to admit four fingers, mucous membrane almost black, covered with a viscid, bloody mucus, closely adhering.

Body of uterus measured fourteen inches in length, and eleven inches across at its widest part, circumference twenty-six inches; being at the same time of a mahogany color throughout, but of deeper color at its right side. Fallopian tubes involved in the discoloration. No external appearance of active inflammation. Parenchyma very soft, and throughout engorged with venous blood, and œdematously infiltrated.

On being opened, the placenta was found to be seated in the right side, and already detached from its insertion. It was a homogeneous, black, and soft mass, difficult to discriminate its structure. At the corresponding portion of the womb the internal surface was exceedingly soft, disintegrated, and more discolored than elsewhere. About two inches above the internal orifice of the same side, the integuments had been, superficially and to the extent of about the size a dime, destroyed. Everywhere hæmorrhagic clots in the parenchyma, even of the oviducts.

No trace of the decidua uterina.

The fœtus is of the female sex, and well matured; I should think it to be about seven or eight months old. It bore every sign of asphyxia.

Lungs extremely hypostatic, dark with a firmer feel. No clot in their respective parenchyma, no thrombus nor embolus in the pulmonary artery.

Heart fat, and the walls of the right ventricle attenuated, rather soft and flabby; of left, hypertrophied. No other diseased condition discernible.

In conclusion, I may state that there was no decomposition, or its attending signs of putrification.

Unfortunately, but little information could be gathered about the previous history of the case; which on account of co-existing circumstances elicited a great deal of newspaper-interest at the time. I learned that the deceased had been a servant; that her pregnancy had been known to her employer; that she had had some intercourse with a woman in South Brooklyn, notorious as an abortionist; and, in fine, that though she had been a sufferer of late, she had done all her housework with usual promptitude up to within three days of her death. Whatever conjecture may be formed from this statement, the remote causes of death remain obscure. The morbid conditions are evidently uniform, and the link of connection cannot be misunderstood. Moreover they cannot have long existed to that extent; as they would have prostrated her, and rendered her incapable of performing the duties of her occupation. Perhaps they were the effects of ergot or other strong emmenagogues; for the injury of the womb indicates the attempt of procuring abortion by mechanical means.

This, however, remains mere conjecture, upon which some light may be thrown by the large experience of other Fellows of the Society.

I wish, however, to draw the attention of the Society to the structural condition of the various organs which were found so extensively diseased; among them the liver is paramount.

I was not at all surprised to find, under the microscope, this organ entirely broken up; its anatomical elements partially disconnected from each other, and only held together by a granulated, hyaline substance, of a very pale yellowish color; but I was surprised to find bodies therein of multangular form, not unlike starch granules, refracting the light strongly, and sometimes acting like a prism in decomposing the light. A watery solution of iodine gave them a dark brown color, which changed into red and violet under the addition of diluted sulphuric acid.

You recollect this to be the reaction of cholesterine; and the same has been stated as to the so-called amyloid degeneration, of late the object of close investigation in Europe. Not depending on my own but inefficient experience with the microscope, I requested Prof. Peaslee to assist me with his superior skill. He has come as nigh as possible to the same results. The same amyloid degeneration, I met with in the kidneys and spleen; although in these organs the amyloid bodies were not of the same size, but rather smaller.

In the heart I found the muscular fibres filled or beset with exceedingly fine, fat globules; and a few amyloid bodies were interspersed. The same structural condition is exhibited by the uterus, which organ I have the honor of presenting to the Society.

Rokitansky first called attention to this peculiar degeneration, which H. von Meckel identifies with the corpora amylacea found by Purkinje in the brain. Meckel, Luschka, Schlossberger and Kölliker suppose them to be a species of fatty degeneration; whereas Virchow, Loewig, Schacht, and Hulley pronounce it to be as near vegetable cellulose as possible; a view to which Rokitansky, also, inclines of late.

Formerly it was supposed that the spleen was the exclusive seat of amyloid degeneration; but Virchow and Traube have since elicited the fact that the liver, the kidneys, and other organs are susceptible of this species of fatty disorganization; and Billroth has lately observed the amyloid bodies in lymphatic glands.

As to the cause of death, I premise that the condition of the liver seriously interfered with the circulation in the cava inferior, while the heart was perfectly unable to propel the blood. The extensive hæmorrhagic extravasations are no doubt solely attributable to the fatty degeneration of the small veins, incapable of bearing the pressure of the stasis,

